# Hepatic accumulation of intestinal cholesterol is decreased and fecal cholesterol excretion is increased in mice fed a high-fat diet supplemented with milk phospholipids

**DOI:** 10.1186/1743-7075-7-90

**Published:** 2010-12-31

**Authors:** Alvin Kamili, Elaine Wat, Rosanna WS Chung, Sally Tandy, Jacquelyn M Weir, Peter J Meikle, Jeffrey S Cohn

**Affiliations:** 1 Nutrition and Metabolism Group, Heart Research Institute, Sydney, Australia; 2 Metabolomics Laboratory, Baker IDI Heart and Diabetes Institute, Melbourne, Australia; 3 Institute of Chinese Medicine, The Chinese University of Hong Kong, Shatin, New Territories, Hong Kong SAR, China

## Abstract

**Background:**

Milk phospholipids (PLs) reduce liver lipid levels when given as a dietary supplement to mice fed a high-fat diet. We have speculated that this might be due to reduced intestinal cholesterol uptake.

**Methods:**

Mice were given a high-fat diet for 3 or 5 weeks that had no added PL or that were supplemented with 1.2% by wt PL from cow's milk. Two milk PL preparations were investigated: a) a PL-rich dairy milk extract (PLRDME), and b) a commercially-available milk PL concentrate (PC-700). Intestinal cholesterol uptake was assessed by measuring fecal and hepatic radioactivity after intragastric administration of [^14^C]cholesterol and [^3^H]sitostanol. Fecal and hepatic lipids were measured enzymatically and by ESI-MS/MS.

**Results:**

Both PL preparations led to significant decreases in total liver cholesterol and triglyceride (-20% to -60%, *P *< 0.05). Hepatic accumulation of intragastrically-administered [^14^C]cholesterol was significantly less (-30% to -60%, *P *< 0.05) and fecal excretion of [^14^C]cholesterol and unlabeled cholesterol was significantly higher in PL-supplemented mice (+15% to +30%, *P *< 0.05). Liver cholesterol and triglyceride levels were positively correlated with hepatic accumulation of intragastrically-administered [^14^C]cholesterol (*P *< 0.001) and negatively correlated with fecal excretion of [^14^C]cholesterol (*P *< 0.05). Increased PL and ceramide levels in the diet of mice supplemented with milk PL were associated with significantly higher levels of fecal PL and ceramide excretion, but reduced levels of hepatic PL and ceramide, specifically, phosphatidylcholine (-21%, *P *< 0.05) and monohexosylceramide (-33%, *P *< 0.01).

**Conclusion:**

These results indicate that milk PL extracts reduce hepatic accumulation of intestinal cholesterol and increase fecal cholesterol excretion when given to mice fed a high-fat diet.

## Background

Dietary phospholipids (PLs) have been shown to reduce plasma and liver lipid levels in experimental animals [1], suggesting that they might be of therapeutic value in patients with hyperlipidemia and/or fatty liver disease. Our laboratory has therefore been investigating the lipid-lowering properties of PL from different sources [2-4], and we have shown that a PL-rich dairy milk extract (PLRDME), containing predominantly phosphatidylcholine (PC), sphingomyelin (SM) and phosphatidylethanolamine (PE), significantly lowers plasma and liver lipid levels in mice fed a high-fat diet. Addition of 2.5% by weight (wt) of milk PL extract to a diet containing 21% butterfat and 0.15% cholesterol resulted in lower levels of serum cholesterol (-23%), and significantly reduced levels of liver triglyceride (-44%) and liver cholesterol (-48%) [2].

Unlike PL from vegetable sources, milk PLs contain a low level of polyunsaturated fatty acids, i.e., they have a polyunsaturated:monounsaturated:saturated fatty acid ratio of 10:30:60. The ability of dietary PL to lower plasma and liver lipid levels in mice fed a high-fat diet is therefore unlikely to be due to their polyunsaturated fatty acid content. This is supported by recent work from our laboratory showing that hydrogenated egg and soy PC, containing only saturated fatty acids (i.e., C16:0 and C18:0), lower liver lipids to a greater extent than non-hydrogenated PC preparations, containing 20% and 70% polyunsaturated fatty acids respectively (i.e., C18:2 and C18:3) [4]. A possible mechanism is that: a) lipid-lowering is dependent on the ability of dietary PL to reduce intestinal cholesterol absorption and b) that PLs containing saturated fatty acids are more efficacious inhibitors of intestinal cholesterol absorption than PLs containing unsaturated fatty acids [5]. The ability of specific types of PL to affect cholesterol absorption has been previously documented, and egg PC containing a higher proportion of saturated fatty acids than soy PC has been shown to be more efficacious in reducing the lymphatic absorption of cholesterol in experimental rats [6]. Milk SM, containing longer and more saturated fatty acids, has in turn been shown to be a more effective inhibitor than egg SM [7]. In vitro, uptake and esterification of cholesterol by human intestinal Caco-2 cells is reduced to a greater extent by milk SM or by dipalmitoyl PC (containing saturated fatty acids) than by egg yolk PC, and in mice, milk SM or dipalmitoyl PC, but not egg PC, has been shown to cause a dose-dependent decrease in cholesterol absorption [8].

The aforementioned results have led us to speculate that PL extracts from milk (containing several species of PL with predominantly saturated fatty acids) might be of therapeutic value in humans. The ability of milk PL extracts to affect intestinal fat metabolism in experimental animals fed an obesogenic/atherogenic diet has not been investigated however, and the extent to which their intestinal effects are related to hepatic lipid-lowering has not been evaluated. The aim of the present study therefore was to determine: a) whether dietary milk PL extracts containing different PL species were able to inhibit intestinal cholesterol absorption in mice fed a high-fat diet, and b) the extent to which these milk PL effects in the intestine were related to their potent ability to reduce cholesterol and triglyceride levels in the liver.

## Methods

### Animals and diets

Four to five-week-old male C57BL/6 mice were obtained from Monash University, Melbourne. They were housed in standard cages (5 mice per cage) at a constant temperature of 21°C with a 12-h light/dark cycle. They were allowed *ad libitum *access to diet and water. After 1 week of acclimatization on normal chow diet, mice (n = 10 per group) were given a high-fat semi-purified diet (HF) with or without added PL. Diets were given for 3, 5 or 8 weeks, depending on the protocol of the experiment. The HF diet contained 21% wt/wt butterfat and 0.15% wt/wt cholesterol. Its composition was as follows (g/kg): casein, 195; DL-methionine, 3; sucrose 341.8; wheat starch, 154; cellulose, 50; clarified butter, 210; calcium carbonate, 17.1; sodium chloride, 2.6; potassium citrate, 2.6; potassium dihydrogen phosphate, 6.9; potassium sulphate, 1.6; AIN93G trace minerals, 1.4; choline chloride (65%), 2.5; vitamins, 10; cholesterol, 1.5. PL-supplemented diets (HFPL) were prepared by adding either 2.5% (wt/wt) phospholipid-rich dairy milk extract (PLRDME) or 2% (wt/wt) commercially-prepared phospholipid concentrate 700 (PC-700) to the HF diet. These additions resulted in diets containing equivalent amounts of milk PL (i.e., 1.2% wt/wt). As described previously [2), 57% by wt of PLRDME was fat and 48% of the extract was PL. The major PL components were PE (29%), PC (26%) and SM (22%), with smaller amounts of phosphatidylserine (PS) and phosphatidylinositol (PI). The remaining PL consisted of lysophospholipids. The main fatty acids present were palmitic (16:0), stearic (18:0) and oleic (18:1) acids (i.e., 29%, 10% and 26%, respectively). The polyunsaturated:monounsaturated:saturated fatty acid ratio was 10:30:60. About one-third of the extract by wt was composed of milk protein, principally casein. The PLRDME was provided by MG Nutritionals (Melbourne, Australia), and was stored at 4°C in vacuum sealed bags in the dark. The PC-700 was provided by Fonterra (Palmerston North, New Zealand). Total fat content of the PC-700 was 85% and 7% was lactose. Sixty percent by wt of the extract was PL. This was comprised of 32% PC, 28% PE and 28% SM. Principal fatty acids were: oleic acid (18:1) 31%, palmitic acid (16:0) 21% and stearic acid (18:0) 11%. Food consumption was recorded three times per week and body weight was monitored weekly. Experiments were conducted in conformity with the Public Health Service (PHS) Policy on Humane Care and Use of Laboratory Animals and were approved by the Animal Welfare Committee of the Sydney South West Area Health Service.

### Assessment of intestinal cholesterol absorption

Fecal dual-isotope ratio methodology was used to assess intestinal cholesterol absorption. This methodology is based on the intragastric administration of radiolabeled cholesterol and a radiolabeled phytosterol. It is assumed that the phytosterol is not absorbed in the intestine and is completely excreted in the feces, thus acting as a marker of sterol passage through the intestinal tract. The ratio of the two sterols in the feces is thus used to calculate percentage cholesterol absorption [9]. Radiolabeled sterols were administered in two separate experiments. In the first experiment, groups of mice (n = 10) were given HF and HFPL diets containing PLRDME for two different time periods (i.e., a total of 40 mice were investigated). In the second experiment, an identical protocol was followed except that the PL-supplemented diet contained PC-700 (designated HFPC-700). After 2 or 4 weeks on diet, mice were given a dose of 200 μl olive oil containing 1 μCi [^14^C]cholesterol and 1 μCi [^3^H]sitostanol by intragastric gavage under light methoxyflurane anaesthesia. Mice were then transferred into individual cages with perforated-metal floors. Feces were collected daily for 4 days and stored at -80°C until analysis. Mice were given access to their respective diets during this collection period. At the end of fecal collection, mice were anaesthetized with methoxyflurane and exsanguinated by heart puncture. The total duration of diet feeding was therefore 2 weeks and 4 days, or 4 weeks and 4 days respectively, which for the sake of simplicity were designated as 3- or 5-week dietary periods.

Blood was allowed to clot and serum was separated by centrifugation (1500× g, 10 min). Sera were aliquoted and stored frozen (-80°C) until analysis. Livers were immediately excised, weighed and divided into small pieces (100-150 mg) for storage at -80°C (for lipid and radioactivity analysis). Fecal samples collected for each animal over 4 days were combined and were dried in an oven at 60°C for 24 h. Dried feces were ground to a fine powder using a mortar and pestle. In order to extract total lipid, 100 mg aliquots of the powder and triplicate 10 μl aliquots of the original dosing mixture were added to 1.2 ml of chloroform:methanol (2:1, v/v). Nitrogen-dried lipid extract was 'saponified' in 1 ml of 2N NaOH : methanol (1:1, v/v) and incubated at 60°C for 1 h. Labelled sterols were then extracted into 2 ml of diethyl ether. Duplicate 500 μl aliquots of the organic phase were transferred into scintillation vials and dried. Scintillation fluid (5 ml) was added and radioactivity was counted. Total liver and fecal lipids were determined gravimetrically after extraction with chloroform:methanol. Individual hepatic and fecal lipids were quantitated enzymatically after solubilization in isopropanol. Triglyceride and total cholesterol concentrations were measured by using GPO-PAP and CHOD-PAP kits (Roche Diagnostics), respectively.

### Phospholipid and ceramide analysis by ESI-MS/MS

In brief, lipids were extracted from liver homogenate (12.5 μl of 2 mg/ml protein), 1 mg feces, or 250 μg food samples. Lipids were extracted with chloroform/methanol (2:1; 20 volumes) following the addition of internal standards (100 pmol each of ceramide (Cer) 17:0, glucosylceramide (GC) 16:0 *d3 and *lactosylceramide (DHC) 16:0 *d3 *(Matreya Inc., Pleasant Gap, USA), phosphatidylcholine (PC) 13:0/13:0, phosphatidylethanolamine (PE) 17:0/17:0, phosphatidylserine (PS) 17:0/17:0 and sphingomyelin (SM) 12:0 (Avanti Polar Lipids, Alabaster, USA), together with 1000 pmol each of free cholesterol *(d7) *(Avanti Polar Lipids, Alabaster, USA) and cholesteryl ester (CE) 18:0 *(d6) *(CDN Isotopes, Pointe-Claire, Quebec, Canada).). Samples were vortexed, mixed for 10 min on a rotation mixer, sonicated for 30 minutes and left to stand at room temperature for a further 20 min. Extracts were then centrifuged at 13,000× g for 10 minutes, the supernatant was transferred to a clean tube and dried under nitrogen at 40°C. Lipids were redissolved in 50 μl water saturated BuOH containing 10 mM NH_4_COOH then 50 μl MeOH containing 10 mM NH_4_COOH was added. Analysis was performed by electrospray ionisation-tandem mass spectrometry using a PE Sciex API 4000 Q/TRAP mass spectrometer with a turbo-ionspray source and Analyst 1.5 data system. Prior liquid chromatographic separation was performed on a Zorbax C18, 1.8 μm, 50 × 2.1 mm column at 300 μl/min using the following gradient conditions; 100% A to 0% A over 8.0 min followed by 2.5 min at 0% A, a return to 100% A over 0.5 min then 3.0 min at 100% A prior to the next injection. Solvent A and B consisted of tetrahydrofuran:methanol:water in the ratios (30:20:50) and (75:20:5) respectively, both containing 10 mM NH_4_COOH. Quantification of individual lipid species was performed using scheduled multiple-reaction monitoring (MRM) in positive ion mode. Individual lipid species monitored were the major species (greater than 1% of total) identified in human plasma. MRM experiments were based on product ion of *m/z *264 [sphingosine-H_2_O]^+ ^for Cer, GC and DHC, *m/z *184 [phosphocholine]^+ ^for SM and PC, *m/z *369 [cholesterol-H_2_O]^+ ^for cholesterol and CE and neutral loss (NL) of 141 Da for PE, 185 Da for PS and 277 Da for PI. Each ion pair was monitored for 10-50 ms with a resolution of 0.7 amu at half-peak height and averaged from continuous scans over the elution period. Lipid concentrations were calculated by relating the peak area of each species to the peak area of the corresponding internal standard. PI species were related to the PE internal standard. Total lipids of each class were calculated by summing the individual lipid species.

### Statistical analysis

Values in the text and tables are means ± SEM. Prism 4 for Macintosh (version 4.0c, GraphPad Software, Inc.) was used for statistical analyses. Significant differences between HF and HFPL groups were assessed by Student's *t*-test. Pearson correlation coefficients (r) were determined to assess the relationship between parameters. A probability of *P *< 0.05 was considered to be statistically significant.

## Results

### Metabolic effects of PLRDME supplementation

Metabolic parameters were assessed after 3 or 5 weeks in mice fed the HF diet alone and in those given the HF diet supplemented with PLRDME (Table 1). Three mice had to be excluded because either they did not survive the gavage or they were not metabolically stable following the procedure. Addition of PLRDME to the HF diet had no significant effect on food intake, weight gain or fecal excretion. Liver wt tended to be lower in HFPL vs HF after 3 weeks (11%, *P *= 0.095). This was less evident after 5 weeks (5%, *P *= 0.37). Liver to body wt ratios were however significantly lower in HFPL vs HF after 3 weeks (12%, *P *= 0.02) and tended to be lower after 5 weeks (9%, *P *= 0.06) (Table 1).

**Table 1:** Body weight, liver weight, food intake and fecal excretion of mice fed the high-fat diet alone (HF) or the high-fat diet supplemented with milk phospholipids (HFPL) in the form of phospholipid-rich dairy milk extract  (PLRDME)

	3 weeks		5 weeks	
	HF	HFPL	HF	HFPL
	(n = 10)	(n = 8)	(n = 9)	(n = 10)
Initial body wt (g)	17.6 ± 0.5	17.1 ± 0.9	18.0 ± 0.5	17.6 ± 0.8
Final body wt (g)	23.6 ± 0.5	23.8 ± 0.6	27.7 ± 0.6	28.8 ± 0.8
Wt gain (g)	6.0 ± 0.3	6.7 ± 0.6	9.7 ± 0.7	11.3 ± 0.5
Liver wt (g)	1.20 ± 0.04	1.07 ± 0.06	1.35 ± 0.06	1.29 ± 0.04
Liver wt/body wt (g/100 g)	5.07 ± 0.17	4.48 ± 0.15 *	4.90 ± 0.20	4.47 ± 0.09
Food intake (g/mouse/day)	3.5 ± 0.3	3.2 ± 0.1	2.9 ± 0.1	3.1 ± 0.1
Fecal excretion (g/mouse/day)	0.23 ± 0.02	0.26 ± 0.01	0.23 ± 0.01	0.22 ± 0.01

Values represent means ± SEM.  Significant difference between HF and HFPL groups by Student's t-test: * P < 0.05

Total lipid, cholesterol and triglyceride were significantly lower in the livers of HFPL vs HF mice (-25%, -21% and -35%, respectively) after 3 weeks on diet (Table 2). The lipid-lowering effect of PLRDME was more pronounced after 5 weeks of feeding (i.e., -41%, -39%, and -47%, respectively). When data were combined, as shown on the right of Table 2, PLRDME supplementation was on average associated with a one-third reduction in hepatic lipids. Hepatic accumulation of intragastrically-administered [^14^C]cholesterol was also significantly lower in HFPL vs HF mice (-45% and -34% at 3 and 5 weeks, respectively), which was associated with an increase in fecal [^14^C]cholesterol excretion. When percentage cholesterol absorption was calculated however, no significant differences were observed. At 3 weeks, percentage cholesterol absorption was 52.7 ± 3.7% and 48.4 ± 4.4%, and at 5 weeks it was 55.8 ± 3.1 and 56.9 ± 2.7% for HF vs HFPL mice, respectively. This was due to an unexpected increase in [^3^H]sitostanol in the feces of HFPL vs HF mice, particularly at 3 weeks. Hepatic accumulation of intragastrically-administered [^3^H]sitostanol was in turn lower in HFPL vs HF mice, although this was not statistically significant at 3 weeks. In contrast, total fecal lipid and cholesterol excretion were increased in HFPL vs HF animals, particularly at 3 weeks. No significant differences were observed in fecal triglyceride excretion. Overall, the combined data (right-hand side of Table 2) showed that PLRDME supplementation resulted in a significant increase in the excretion of intragastrically-administered cholesterol and plant sterol (by 30% and 25%, respectively). The calculated percentage cholesterol absorption did not change, though this calculation had limited validity considering that differences were observed in the intestinal metabolism of intragastrically-administered [^3^H]sitostanol.

**Table 2:** Effect of dietary milk phospholipids in the form of phospholipid-rich dairy milk extract (PLRDME) on liver and fecal lipid parameters in mice given an intragastric gavage of radioactively labelled cholesterol and sitostanol after 3 or 5 weeks on high-fat diet

	3 weeks			5 weeks			Combined		
	HF	HFPL	*difference*	HF	HFPL	*difference*	HF	HFPL	*difference*
	(n = 10)	(n = 8)		(n = 9)	(n = 10)		(n = 19)	(n = 18)	
Liver									
Total lipid (mg/organ)	118 ± 6	89 ± 10 *	*-25%*	212 ± 19	126 ± 7 ***	*-41%*	162 ± 14	110 ± 7 **	*-32%*
Cholesterol (μmol/organ)	33 ± 1	26 ± 3 *	*-21%*	64 ± 8	39 ± 4 **	*-39%*	48 ± 5	33 ± 3 *	*-31%*
Triglyceride (μmol/organ)	65 ± 5	42 ± 8 *	*-35%*	168 ± 14	90 ± 10 ***	*-47%*	114 ± 14	70 ± 9 *	*-38%*
[^14^C]cholesterol (dpm/organ)	6819 ± 836	3737 ± 948 *	*-45%*	13560 ± 1737	9021 ± 1043 *	*-34%*	10012 ± 1204	6673 ± 943 *	*-33%*
[^3^H]sitostanol (dpm/organ)	317 ± 31	244 ± 52	*-23%*	547 ± 41	370 ± 38 **	*-32%*	426 ± 37	318 ± 34 *	*-25%*
									
Feces									
Total lipid (mg/mouse/day)	6.4 ± 0.6	10.6 ± 0.7 ***	*+66%*	9.2 ± 1.0	10.2 ± 1.1	*+11%*	7.8 ± 0.7	10.4 ± 0.7 *	*+33%*
Cholesterol (μmol/mouse/day)	7.3 ± 0.4	9.1 ± 0.5 *	*+25%*	6.7 ± 0.3	7.4 ± 0.4	*+11%*	7.0 ± 0.3	8.1 ± 0.4 *	*+16%*
Triglyceride (μmol/mouse/day)	0.9 ± 0.1	1.0 ± 0.2	*+11%*	2.6 ± 0.5	2.0 ± 0.4	*-24%*	1.8 ± 0.3	1.6 ± 0.3	*-11%*
[^14^C]cholesterol (dpm/mouse/day)	9195 ± 1187	13454 ± 996 *	*+46%*	8889 ± 795	10245 ± 864	*+15%*	9050 ± 711	11765 ± 740 *	*+30%*
[^3^H]sitostanol (dpm/mouse/day)	12376 ± 1275	17110 ± 712 **	*+38%*	16076 ± 570	18134 ± 777	*+13%*	14129 ± 828	17679 ± 535 **	*+25%*

Results represent means ± SEM. Mice given the high-fat diet alone were designated HF. Mice given the high-fat diet supplemented with 2.5% by wt PLRDME were designated HFPL. Data are provided for animals fed for 3 or 5 weeks. Combined data are shown on the right. Percentage difference between groups is given in italics. Significant difference between HF and HFPL by Student's t-test: * *P *< 0.05, ** *P *< 0.01, *** *P *< 0.001.

### Metabolic effects of PC-700 supplementation

In order to confirm the effect of milk PL on intestinal cholesterol and plant sterol uptake, a second milk PL preparation was investigated (i.e., PC-700). This commercially-available milk PL concentrate contained more total fat (85% vs 57% by wt) and more PL (60% vs 48% by wt) than PLRDME. Less PC-700 was therefore added to the HF diet, to ensure that the HFPL diets contained comparable amounts of milk PL (i.e.,1.2% by wt). The PC-700 and the PLRDME had a similar PL composition (comprising primarily PC, PE and SM) and a similar fatty acid composition (comprising predominantly palmitic, stearic and oleic acids).

Mice were fed the HF diet and the HF diet containing PC-700 (designated the HFPC-700 group). They were studied in the same way as in the first experiment. Metabolic parameters for these animals are given in Table 3. Two mice had to be excluded from the analysis since they did not react well to the intragastric gavage. Addition of PC-700 to the HF diet had no significant effect on any of the parameters. The tendency of PLRDME to reduce liver weight was not evident in mice supplemented with PC-700.

**Table 3:** Body weight, liver weight, food intake, and fecal excretion of mice fed the high-fat diet alone (HF) or the high-fat diet supplemented with milk phospholipids (HFPC-700) in the form of commercially-prepared PC-700

	3 weeks		5 weeks	
	HF	HFPC-700	HF	HFPC-700
	(n = 10)	(n = 9)	(n = 9)	(n = 10)
Initial body wt (g)	17.0 ± 0.8	17.1 ± 0.6	16.6 ± 0.6	17.0 ± 0.4
Final body wt (g)	25.3 ± 0.5	24.6 ± 0.6	28.7 ± 0.7	29.0 ± 0.9
Wt gain (g)	8.3 ± 0.8	7.5 ± 0.7	12.1 ± 0.9	12.0 ± 0.9
Liver wt (g)	1.14 ± 0.05	1.12 ± 0.04	1.21 ± 0.08	1.12 ± 0.06
Liver wt/body wt (g/100 g)	4.54 ± 0.25	4.58 ± 0.19	4.24 ± 0.30	3.90 ± 0.24
Food intake (g/mouse/day)	3.1 ± 0.1	3.1 ± 0.1	3.2 ± 0.1	3.2 ± 0.1
Fecal mass (g/mouse/day)	0.31 ± 0.01	0.34 ± 0.01	0.35 ± 0.02	0.35 ± 0.02

Values represent means ± SEM.  No significant differences were observed between HF and HFPC-700 groups

Total lipid, cholesterol and triglyceride levels were lower in the livers of HFPC-700 vs HF mice (-22%, -45% and -35%, respectively) after 3 weeks on diet, although this reached statistical significance only for liver cholesterol (Table 4). As in the first experiment with PLRDME, the lipid-lowering effect of PC-700 was more pronounced after 5 weeks of feeding (i.e., -45%, -57%, and -63%, respectively). Hepatic accumulation of intragastrically-administered [^14^C]cholesterol was also significantly lower in HFPC-700 vs HF mice (-60% and -65% at 3 and 5 weeks, respectively). Intestinal absorption of [^3^H]sitostanol was once again evidenced by the presence of intragastrically-administered [^3^H]sitostanol in the liver. It was lower in HFPC-700 vs HF mice, reaching statistical significance when 3-and 5-week data were combined (as shown on the right in Table 4). The combined data demonstrated that PC-700 supplementation was associated with an approximate 30% to 65% reduction in hepatic lipid parameters, which was similar in magnitude to that obtained with the PLRDME. It should be noted however that experiments with PLRDME and PC-700 were not conducted simultaneously and it was therefore difficult to directly compare the efficacy of the two PL preparations.

**Table 4:** Effect of dietary milk phospholipids in the form of commercially-prepared PC-700 on liver and fecal lipid parameters in mice given an intragastric gavage of radioactively-labelled cholesterol and sitostanol after 3 or 5 weeks on high-fat diet.

	3 weeks			5 weeks			Combined		
	HF	HFPC-700	*difference*	HF	HFPC-700	*difference*	HF	HFPC-700	*difference*
	(n = 10)	(n = 9)		(n = 9)	(n = 10)		(n = 19)	(n = 19)	
Liver									
Total lipid (mg/organ)	103 ± 10	80 ± 5	-22%	148 ± 18	82 ± 7 **	-45%	124 ± 11	81 ± 4 **	-35%
Cholesterol (μmol/organ)	30 ± 3	17 ± 1 **	-45%	37 ± 4	16 ± 2 ***	-57%	34 ± 3	16 ± 1 ***	-51%
Triglyceride (μmol/organ)	80 ± 13	52 ± 8	-35%	110 ± 14	41 ± 8 ***	-63%	94 ± 10	46 ± 6 ***	-51%
[^14^C]cholesterol (dpm/organ)	5377 ± 902	2150 ± 248 **	-60%	6390 ± 1006	2268 ± 305 ***	-65%	5857 ± 664	2212 ± 194 ***	-62%
[^3^H]sitostanol (dpm/organ)	403 ± 76	264 ± 31	-34%	310 ± 49	206 ± 28	-33%	359 ± 46	234 ± 21 *	-35%
									
Feces									
Total lipid (mg/mouse/day)	15.5 ± 1.4	22.3 ± 1.7 **	+44%	12.3 ± 1.1	18.8 ± 2.0 *	+53%	14.0 ± 1.0	20.4 ± 1.3 ***	+46%
Cholesterol (μmol/mouse/day)	6.8 ± 0.7	7.9 ± 0.6	+17%	6.8 ± 0.5	8.4 ± 0.5 *	+24%	6.8 ± 0.4	8.2 ± 0.4 *	+21%
Triglyceride (μmol/mouse/day)	4.6 ± 1.1	6.1 ± 1.5	+33%	2.3 ± 0.6	3.7 ± 1.0	+62%	3.5 ± 0.7	4.9 ± 0.9	+38%
[^14^C]cholesterol (dpm/mouse/day)	10289 ± 675	12163 ± 437 *	+18%	9075 ± 770	11208 ± 550 *	+24%	9714 ± 515	11660 ± 364 **	+20%
[^3^H]sitostanol (dpm/mouse/day)	17778 ± 962	19490 ± 981	+10%	18173 ± 907	17552 ± 618	-3%	17965 ± 647	18470 ± 596	+3%

Results represent means ± SEM. Mice given the high-fat diet alone were designated HF. Mice given the high-fat diet supplemented with 2% by wt PC-700 were designated HFPC-700. Data are provided for animals fed for 3 or 5 weeks. Combined data are shown on the right. Percentage difference between groups is given in italics. Significant difference between HF and HFPC-700 by t-test: * *P *< 0.05, ** *P *< 0.01, *** *P *< 0.001.

Total fecal lipid and cholesterol excretion were in contrast increased in HFPC-700 vs HF animals. No significant differences were observed in fecal triglyceride excretion, although the combined data suggested an increase. Excretion of intragastrically-administered [^14^C]cholesterol was also increased in HFPC-700 mice. Unlike the results of the first experiment, excretion of intragastrically-administered [^3^H]sitostanol was not significantly higher in HF mice supplemented with PC-700. Thus calculated percentage cholesterol absorption was lower in HFPL vs HF mice: 36.7 ± 2.0% vs 44.5 ± 2.4% at 3 weeks (*P *= 0.03) and 37.9 ± 2.0% vs 52.1 ± 3.1% at 5 weeks (*P *= 0.001). The validity of this calculation must be questioned however in view of the apparent intestinal uptake of [^3^H]sitostanol (as noted in the first experiment). The combined data showed that PC-700 supplementation resulted in a significant increase in fecal excretion of total lipid, unlabelled cholesterol and radioactively-labelled intragastrically-administered cholesterol (Table 4).

### Liver lipid levels and intestinal uptake of cholesterol

**Figure 1:**
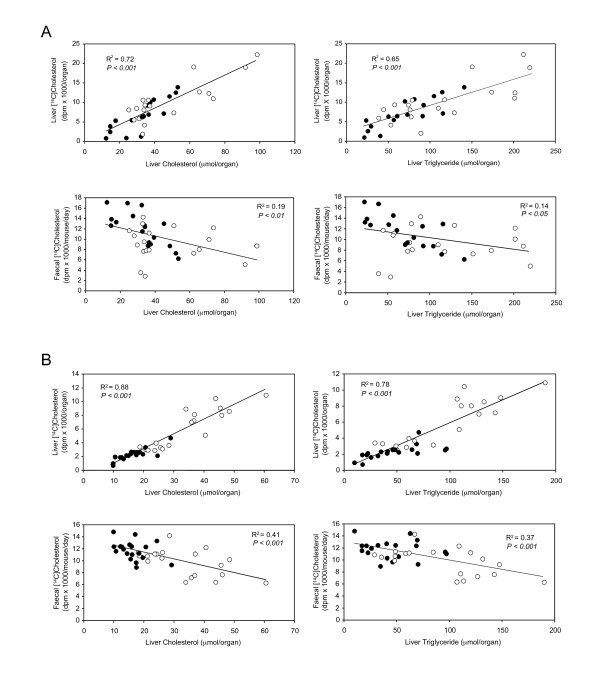
**Relationship between liver lipids and intragastrically-administered [^14^C]cholesterol in the liver and feces of mice fed the high-fat diet alone or the high-fat diet supplemented with milk phospholipids: (A) PLRDME or (B) PC-700**. Data points represent individual animals after 3 or 5 weeks of diet feeding. Open circles represent high-fat-fed mice (HF); filled circles represent milk phospholipid-supplemented animals (HFPL or HFPC-700). A correlation coefficient (r^2^) is shown in each panel together with its level of statistical significance.

Linear regression analysis was carried out in order to determine whether there was any relationship between liver lipid levels and the distribution of intragastrically-administered [^14^C]cholesterol between the liver and feces of diet-fed animals. Results for PLRDME-supplemented mice are shown in Figure 1A, and those for PC-700-supplemented mice are shown in Figure 1B. In both cases, a strong positive correlation was found between liver cholesterol and triglyceride levels and the hepatic accumulation of intragastrically-administered [^14^C]cholesterol. At the same time, a negative correlation was found between liver cholesterol and triglyceride levels and fecal excretion of intragastrically-administered [^14^C]cholesterol.

### Further analysis of fecal lipids in mice supplemented with PLRDME

Conversion of fecal cholesterol and triglyceride measurements to mg units revealed that only one third of total fecal lipid was accounted for by cholesterol and triglyceride. Thin layer chromatography was therefore carried out to determine what other lipids were present and it was found that fecal samples contained appreciable amounts of fatty acid and in the case of PL-supplemented animals, high levels of PL and ceramides. In order to measure these latter lipids by ESI-MS, fecal samples without radioactivity were required and a third experiment was carried out in which one group of mice (n = 10) were fed the HF diet and a second group (n = 10) was fed the HF diet supplemented with PLRDME. Mice were fed for 8 weeks and 24-h fecal samples were collected at 2 (n = 3), 4 (n = 4) and 8 weeks (n = 4) from each cage of animals (n = 5 mice per cage). After 8 weeks, liver wt was not significantly different in HF vs HFPL (1.65 ± 0.06 vs 1.44 ± 0.08 g, respectively), however when expressed as a ratio to body wt., HFPL mice had significantly smaller livers (4.32 ± 0.23 vs 5.15 ± 0.15 g/100 g, *P *< 0.05). Total lipid was also significantly reduced in the liver of HFPL animals (12.3 ± 0.4 vs 15.1 ± 1.4 g/100 g, *P *< 0.05), predominantly due to a reduction in hepatic triglyceride (116.9 ± 6.6 vs 162.2 ± 23.2 μmol/g, *P *< 0.05). Plasma cholesterol but not plasma triglyceride was significantly lower in HFPL mice (4.7 ± 0.4 vs 6.1 ± 0.3, *P *< 0.05 and 0.71 ± 0.06 vs 0.56 ± 0.07 mmol/l, n.s). These results demonstrated that PLRDME supplementation reduced both hepatic and serum lipid levels in this group of animals. Fecal PL and ceramide levels were analysed by ESI-MS and fecal excretion was calculated in units of nmol of lipid per mouse per day (Table 5). In HF mice the predominant PL species in feces were PC, lysoPC and SM. In HFPL mice the predominant species were PC and SM. All PL species were significantly higher in the feces of HFPL vs HF animals. PC was increased 2.8-fold, PI was increased 6.2-fold and SM was increased 5.6-fold. The major PC species (in descending order of abundance) and their fecal excretion rates (nmol/mouse/day) in HF and HFPL mice respectively were: PC 32:1, 18.5 ± 1.6 vs 59.3 ± 5.0, *P *< 0.001; PC 34:1, 17.8 ± 2.2 vs 35.8 ± 3.6, *P *< 0.001; PC 32:0, 6.0 ± 0.7 vs 44.7 ± 4.2, *P *< 0.001; PC 34:0, 3.0 ± 0.3 vs 43.2 ± 4.0, *P *< 0.001. The major SM species (in descending order of abundance) and their fecal excretion rates (nmol/mouse/day) in HF and HFPL mice were: SM 24:1, 30.7 ± 2.8 vs 138.9 ± 12.5, *P *< 0.001; SM 16:0, 14.9 ± 1.7 vs 54.5 ± 6.3, *P *< 0.001; SM 22:0, 3.8 ± 0.4 vs 97.1 ± 9.6, *P *< 0.001; SM 24:0, 1.9 ± 0.2 vs 54.2 ± 4.7, *P *< 0.001. All ceramide species were also significantly higher in the feces of HFPL vs HF animals (i.e., 4.2-fold, 9.3-fold and 10.2-fold for ceramide (Cer), monohexosylceramide (MHC) and dihexosylceramide (DHC), respectively). The major ceramide species (in descending order) and their fecal excretion rates (nmol/mouse/day) in HF and HFPL mice were: Cer 16:0, 11.1 ± 1.0 vs 41.6 ± 3.4, *P *< 0.001; Cer 18:1, 7.4 ± 0.9 vs 31.5 ± 2.7, *P *< 0.001; Cer 18:0, 4.2 ± 0.4 vs 20.4 ± 1.7, *P *< 0.001; Cer 22:0, 2.4 ± 0.2 vs 26.3 ± 2.6, *P *< 0.001; Cer 24:0, 1.9 ± 0.2 vs 23.2 ± 2.4, *P *< 0.001. The three most abundant MHC and DHC species (in descending order) and their fecal excretion rates (nmol/mouse/day) in HF and HFPL mice respectively were: DHC 22:0, 8.1 ± 0.4 vs 89.4 ± 5.1, *P *< 0.001; DHC 24:0, 4.9 ± 0.2 vs 87.5 ± 4.9, *P *< 0.001; DHC 16:0, 4.1 ± 0.2 vs 49.7 ± 3.6, *P *< 0.001; MHC 22:0, 3.5 ± 0.3 vs 39.1 ± 3.2, *P *< 0.001; MHC 24:0, 2.0 ± 0.1 vs 36.1 ± 3.0, *P *< 0.001; MHC 16:0, 1.7 ± 0.1 vs 13.4 ± 1.3, *P *< 0.001. We confirmed that fecal cholesterol excretion was increased in mice supplemented with PLRDME (as in the first study) and found that this was due to a difference in free cholesterol excretion (HFPL vs HF: 3344 ± 329 vs 2486 ± 228 nmol/day/mouse, *P *< 0.05). Interestingly, fecal cholesteryl ester (CE) excretion was significantly lower in HFPL mice. The most abundant CE species and their fecal excretion rates (nmol/mouse/day) in HF and HFPL mice respectively were: CE 16:0, 32.2 ± 2.4 vs 26.3 ± 1.6, *P *= 0.05*; *CE 18:1, 19.9 ± 1.4 vs 17.8 ± 1.2, *P *= 0.26, n.s; CE 16:1, 16.4 ± 2.0 vs 10.8 ± 1.1, *P *< 0.05; CE 18:2, 14.4 ± 1.6 vs 10.5 ± 1.7, *P *= 0.11, n.s. ESI-MS measurements of fecal triglyceride and diglyceride species confirmed that these lipids were not significantly different between groups.

**Table 5:** Phospholipid and ceramide levels in the feces of mice fed the high-fat diet alone (HF) or the high-fat diet supplemented with milk phospholipids (HFPL) in the form of phospholipid-rich dairy milk extract (PLRDME)

	HF	HFPL	*difference*
*nmol/mouse/day*			
Phosphatidylcholine	71 ± 7	272 ± 22 ***	*+281%*
Lysophosphatidylcholine	60 ± 7	92 ± 8 **	*+52%*
Phosphatidylethanolamine	7 ± 1	13 ± 1 **	*+79%*
Phosphatidylinositol	3.1 ± 0.4	22.5 ± 2.6 ***	*+617%*
Phosphatidylserine	3.3 ± 0.3	4.8 ± 0.4 *	*+43%*
Sphingomyelin	55 ± 5	362 ± 34 ***	*+560%*
			
Ceramide	29 ± 2	150 ± 13 ***	*+424%*
Monohexosylceramide	10 ± 1	100 ± 8 ***	*+934%*
Dihexosylceramide	23 ± 1	258 ± 15 ***	*+1017%*
Trihexosylceramide	0.8 ± 0.1	2.5 ± 0.2 ***	*+212%*

Results represent means ± SEM for 11 fecal samples in each group. Percentage difference between groups is given in italics. Significant difference between HF and HFPL by Student's t-test: * *P *< 0.05, ** *P *< 0.01, *** *P *< 0.001.

### Analysis of hepatic phospholipids in mice supplemented with PLRDME

In view of the higher levels of PL in the diet and in the feces of HFPL mice, we questioned whether this PL was associated with altered levels of hepatic PL (i.e., concomitant with the observed decreases in hepatic triglyceride and cholesterol). PL was measured directly in duplicate samples of whole diet, and intake of individual PL species was calculated for mice in the 8-week study (i.e., the third experiment) in terms of nmol of PL consumed per mouse per day. PL species were measured in the liver of mice sacrificed after 8 weeks on diet. All PL species (with the exception of lysophospholipid) were increased about 40-fold in the HFPL diet. Liver PL levels were not increased however and in fact tended to be lower in HFPL vs HF mice. Liver PC (the major PL species in the liver) was significantly less in HFPL vs HF livers (-21%, *P *< 0.05). The most abundant hepatic PL species (in descending order) and their levels (nmol/liver) in HF and HFPL mice were: PC 34:1, 1623 ± 101 vs 1223 ± 125, -25%, *P *< 0.05; PC 32:1, 1280 ± 140 vs 1029 ± 72, -20%, *P *= 0.12, ns; PC 34:2, 902 ± 110 vs 677 ± 57, -25%, *P *= 0.08; PC 38:6, 694 ± 54 vs 537 ± 44, -23%, *P *< 0.05; PC 36:3, 551 ± 47 vs 395 ± 29, -28%, *P *< 0.05. The most abundant dietary PL species consumed by the HF and HFPL mice, respectively, were (in nmol/mouse/day): PC 32:1, 91 vs 3175; PC 34:1, 45 vs 2525; PC 36:2, 38 vs 1051; and PC 32:0, 24 vs 1025. Ceramide intake was also higher in HFPL mice (Table 6), particularly MHC and DHC, however hepatic ceramide levels tended to be lower. Total liver Cer was lower by 22% in HFPL mice (P = 0.07). The most abundant Cer species in the liver (in descending order) and their level in HF and HFPL mice were (in nmol/liver): Cer 24:1, 24 ± 2 vs 15 ± 1, -36%, *P *< 0.01; Cer 22:0, 20 ± 2 vs 16 ± 1, -17%, *P *= 0.23; Cer 24:0, 12 ± 2 vs 11 ± 1, -10%, *P *= 0.49; Cer 16:0, 10 ± 1 vs 9 ± 1, -13%, *P *= 0.27. The most abundant MHC species in the liver (in descending order) and their level in HF and HFPL mice were (in nmol/liver): MHC 22:0, 9.9 ± 1.0 vs 6.2 ± 0.6, -37%, *P *< 0.01; MHC 24:0, 5.3 ± 0.3 vs 3.9 ± 0.2, -26%, *P *< 0.01; MHC 24:1, 5.3 ± 0.7 vs 3.6 ± 0.3, -32%, *P *< 0.05.

**Table 6:** Phospholipid and ceramide levels in the food and liver of mice fed the high-fat diet alone (HF) or the high-fat diet supplemented with milk phospholipids in the form of phospholipid-rich dairy milk extract (PLRDME)

	Food Intake			Liver		
	HF	HFPL	*difference*	HF	HFPL	*difference*
	*nmol/mouse/day*			*nmol/whole organ*		
Phosphatidylcholine	279	11382	*+3980%*	7568 ± 620	5947 ± 412 *	*-21%*
Phosphatidylethanolamine	270	12963	*+4701%*	2022 ± 152	1814 ± 99	*-10%*
Phosphatidylinositol	72	3242	*+4403%*	1211 ± 95	1019 ± 68	*-16%*
Phosphatidylserine	136	5002	*+3613%*	141 ± 10	129 ± 6	*-8%*
Lysophosphatidylcholine	662	1048	*+58%*	274 ± 92	222 ± 28	*-19%*
Sphingomyelin	245	9670	*+3847%*	739 ± 71	622 ± 20	*-16%*
						
Ceramide	41	87	*+112%*	75 ± 7	58 ± 4	*-22%*
Monohexosylceramide	29	858	*+857%*	24 ± 2	16 ± 1 **	*-33%*
Dihexosylceramide	45	1295	*+1294%*	2.7 ± 0.2	2.4 ± 0.2	*-11%*
Trihexosylceramide	5	6	*+20%*	nd	nd	*nd*

Food intake data represent averages of duplicate measurements. Liver lipid results represent means ± SEM for 8 mice in each group. Percentage difference between groups is given in italics. Significant difference between HF and HFPL by Student's t-test: * *P *< 0.05, ** *P *< 0.01, *** *P *< 0.001.

## Discussion

The present data support our previous work showing that the addition of 1.2% by wt milk PLs to the diet reduces hepatic cholesterol and triglyceride levels in mice fed a high-fat diet [2]. Reduced lipid levels in the liver of PL-supplemented mice were associated with a reduction in hepatic accumulation of intragastrically-administered [^14^C]cholesterol and an increase in fecal cholesterol excretion. These results suggest that one of the mechanisms whereby milk PL extracts beneficially reduce elevated hepatic lipid levels in mice fed an obesogenic and/or atherogenic diet is by reducing intestinal cholesterol uptake.

A number of different mechanisms can be proposed to explain how dietary milk PL interferes with intestinal cholesterol uptake. Firstly, it has been shown that pancreatic PLA_2 _hydrolysis of luminal PL is an important prerequisite for intestinal cholesterol absorption [10,11] and that excess PL can inhibit PLA_2_-dependent cholesterol uptake [12]. Secondly, PL has a strong effect on cholesterol solubilization in intestinal micelles [8] and excess luminal PL may affect solubilization, thus shifting the partition coefficient of cholesterol away from the enterocyte membrane. A third possibility is that increased PL concentration in the gut lumen results in increased size and altered physical characteristics of lipid micelles, thereby affecting the interaction of cholesterol with specific protein transporters responsible for intestinal transport [13]. A final possibility is that excess luminal PL is able to stimulate cholesterol excretion in the proximal part of the small intestine - a pathway that has been termed: "transintestinal cholesterol efflux" or TICE [14]. Both bile salts and PLs have been shown in intestinal perfusion studies in mice to stimulate TICE [15]. Support for this mechanism was unexpectedly obtained in our first experiment in which mice were supplemented with PLRDME. Fecal excretion of [^3^H]sitostanol was increased in PLRDME-supplemented animals (Table 2). After 8 weeks on diet, gene expression of ABCG5 and ABCG8 in the proximal intestine (proteins mediating plant sterol excretion from the enterocyte into the gut lumen) was also found to be significantly increased (data not shown). Similar results were not however obtained in the second experiment with PC-700 (Table 4), suggesting that increased TICE was not sufficient to explain increased sterol excretion in PL-supplemented animals.

Previous studies have provided evidence that dietary PLs have the ability to reduce intestinal cholesterol absorption [5]. We therefore set out to calculate percentage cholesterol absorption in the present study using a fecal dual-isotope method that has been validated and used extensively in mice [9]. This indirect method for calculating percentage cholesterol absorption relies on the assumption that intragastrically-administered [^3^H]sitostanol is not absorbed. Unexpectedly, this was not the case in the present experiments, as evidenced by the presence of [^3^H]sitostanol in the liver of diet-fed mice. Calculated percentages of absorbed cholesterol in the present study were therefore unreliable and they should be interpreted with caution.

Two studies have investigated the acute effects of milk SM administered intragastrically to rats [7,16], while a third involved feeding milk SM to mice over 4 days [8]. In this latter study, a dose of 0.1% by wt SM added to normal dietary chow was found to reduce cholesterol absorption by 20%. The physiological and/or therapeutic relevance of this effect was not however investigated. In the present experiments, milk PL extracts were fed for 3 or 5 weeks to mice fed a diet that induces many of the atherogenic features characteristic of the metabolic syndrome in humans [17]. PL supplementation resulted in a 20% to 60% decrease in hepatic cholesterol and a 15% to 30% increase in fecal cholesterol excretion (Tables 2 and 4). Milk PL extracts were added to the high-fat diet at a level of 1.2% by wt PL and the level of SM in PLRDME and PC-700 was 22% and 28% of total PL, respectively. The HFPL and HFPC-700 diets thus contained about 0.3% by wt milk SM. Comparing this level to that used in the short-term study by Eckhardt *et al*. (i.e., 0.1%) [8], it can be proposed that the amount of SM in our milk extracts may have been sufficient to account for the observed intestinal effects. Other PL species may also have contributed however - a possibility supported by studies showing that lecithin or PC from different sources can affect intestinal cholesterol absorption [6,18,19].

It is generally believed that increased flux of cholesterol from the intestine to the liver, due to increased dietary cholesterol, results in increased production of oxysterols in the liver and activation of the LXR-SREBP-1c pathway [20,21]. Fatty acid synthesis is enhanced, promoting the production of hepatic triglycerides and the development of liver steatosis. Reduced intestinal cholesterol uptake therefore tends to have the opposite effect, and this is a likely explanation for the ability of dietary milk PLs to lower hepatic triglyceride levels. Support for this scenario comes from studies with the cholesterol absorption inhibitor ezetimibe, which has been shown to reduce hepatic LXR activity and reduce hepatic steatosis [22]. In previous experiments, we have found that PLRDME supplementation causes a significant decrease in LXR-regulated genes as well as the expression of enzymes affecting fatty acid synthesis (e.g., fatty acid synthase and acetyl-CoA carboxylase) [2]. Additional work involving the direct measurement of hepatic TG synthesis is nevertheless required to substantiate the link between reduced cholesterol absorption and reduced hepatic TG levels in PL-supplemented mice.

Supplementation of the HF diet with 2.5% by wt PLRDME (i.e.,1.2% by wt PL) resulted in an approximately 40-fold increase in the amount of individual PLs in the diet (Table 6). This resulted in a significant increase in fecal excretion of all PL species, however the magnitude of the increase was much less than in the diet, e.g., fecal excretion increased only 2.8-, 5.6-, 0.8- and 6.2-fold for PC, SM, PE and PI, respectively. Comparing the food intake data in Table 6 (expressed as nmol per mouse per day) with the fecal excretion data in Table 5 (expressed in the same units), it can be calculated that 2.4%, 3.7%, 0.1% and 0.2% of PC, SM, PE and PI in the diet appeared in the feces, which indicates that the added PL was very efficiently absorbed. Interestingly, increased PL absorption was not associated with a significant increase in plasma PL levels (data not shown) nor in levels of hepatic PLs (Table 6). In fact, liver PL levels tended to be lower and liver PC was significantly reduced (i.e., by 21%) in PLRDME-supplemented mice. Liver ceramide levels also tended to be lower, despite increased amounts of dietary and fecal ceramide. Yunoki *et. al*. have recently shown that glucosylceramide from corn (added to the diet at a level of 0.5% by wt) reduced fatty liver in Zucker fatty rats [23]. Duivenvoorden *et. al*. have documented the lipid-lowering activity of dietary sphingolipids (including yeast ceramide) in genetically-induced hyperlipidemic mice [24]. Furthermore, Feng *et. al*. have recently demonstrated that ceramide inhibits cholesterol uptake to a greater extent than SM in cultured human intestinal cells [25]. Milk sphingolipids, including milk ceramides, may therefore have contributed to the lipid-lowering effects observed in the present study. This potentially beneficial effect of dietary ceramides is in stark contrast to the detrimental role of endogenous ceramide biosynthesis that has been proposed to be of central importance in the etiology of obesity, insulin resistance and the metabolic syndrome [26,27].

The current results suggest that milk PL extracts might be of therapeutic benefit in individuals with fatty liver disease or those with hyperlipidemia. The extent to which dietary PLs can affect plasma and liver lipid levels remains to be determined however. Human studies [28-30] carried out in a small number of individuals support the concept that luminal or dietary lecithin (i.e., PC) can reduce intestinal cholesterol absorption in man. In one of these studies, human subjects were infused intraduodenally with a relatively large amount of lecithin (150 mg/kg/h), which not only blocked cholesterol absorption but actually "extracted" cholesterol from the intestinal mucosa, reflected by 'negative' absorption values [28]. In a second study, 10 hypertriglyceridemic patients were treated with lecithin (10 g/day), and after 2-3 weeks, cholesterol absorption was reduced from 42 ± 2% to 36 ± 2% (*P *< 0.05) [30]. The ability of milk PL extracts to mimic and/or potentiate this effect deserves further investigation.

## Conclusions

The present experiments demonstrate that milk PL extracts have beneficial effects on liver and intestinal cholesterol metabolism in mice fed an obesogenic and/or atherogenic diet. More specifically, the present work provides evidence that milk PL extracts have the potential to reduce intestinal cholesterol uptake. Whether or not this is of therapeutic relevance in man remains to be determined.

## List of abbreviations

HF: high-fat diet group; HFPC-700: high-fat diet group supplemented with PC-700; HFPL: high-fat diet group supplemented with PLRDME; PC: phosphatidylcholine; PE: phosphatidylethanolamine; PI: phosphatidylinositol; PL: phospholipid; PLRDME: phospholipid-rich dairy milk extract; PS: phosphatidylserine; SM: sphingomyelin

## Competing interests

PLRDME used in the present study was kindly provided by MG Nutritionals, Melbourne, Australia and PC-700 was provided by Fonterra, Palmerston North, New Zealand. These companies did not otherwise contribute to this work.

## Authors' contributions

AK carried out all aspects of the present study including the design, the feeding of animals, collection and analysis of samples, data analysis and the preparation of the manuscript. EW, RC and ST helped in the maintenance of animals, collection and analysis of samples and in the review of the final manuscript. JW and PM were instrumental in carrying out the ESI-MS analysis. JC was involved in the design and implementation of all experiments, data analysis and writing. All authors read and approved the final manuscript.
